# Exploring the Seasonality of Reported Treated Malaria Cases in Mpumalanga, South Africa

**DOI:** 10.1371/journal.pone.0076640

**Published:** 2013-10-29

**Authors:** Sheetal Prakash Silal, Karen I. Barnes, Gerdalize Kok, Aaron Mabuza, Francesca Little

**Affiliations:** 1 Department of Statistical Sciences, University of Cape Town, Cape Town, South Africa; 2 Division of Clinical Pharmacology, Department of Medicine, University of Cape Town, Cape Town, South Africa; 3 Malaria Elimination Programme, Mpumalanga Department of Health, Nelspruit, South Africa; Johns Hopkins University, United States of America

## Abstract

South Africa, having met the World Health Organisation's pre-elimination criteria, has set a goal to achieve malaria elimination by 2018. Mpumalanga, one of three provinces where malaria transmission still occurs, has a malaria season subject to unstable transmission that is prone to sporadic outbreaks. As South Africa prepares to intensify efforts towards malaria elimination, there is a need to understand patterns in malaria transmission so that efforts may be targeted appropriately. This paper describes the seasonality of transmission by exploring the relationship between malaria cases and three potential drivers: rainfall, geography (physical location) and the source of infection (local/imported). Seasonal decomposition of the time series by Locally estimated scatterplot smoothing is applied to the case data for the geographical and source of infection sub-groups. The relationship between cases and rainfall is assessed using a cross-correlation analysis. The malaria season was found to have a short period of no/low level of reported cases and a triple peak in reported cases between September and May; the three peaks occurring in October, January and May. The seasonal pattern of locally-sourced infection mimics the triple-peak characteristic of the total series while imported infections contribute mostly to the second and third peak of the season (Christmas and Easter respectively). Geographically, Bushbuckridge municipality, which exhibits a different pattern of cases, contributed mostly to the first and second peaks in cases while Maputo province (Mozambique) experienced a similar pattern in transmission to the imported cases. Though rainfall lagged at 4 weeks was significantly correlated with malaria cases, this effect was dampened due to the growing proportion of imported cases since 2006. These findings may be useful as they enhance the understanding of the current incidence pattern and may inform mathematical models that enable one to predict the impact changes in these drivers will have on malaria transmission.

## Introduction

Despite being a treatable and preventable mosquito-borne disease, malaria is still an immense global health, economic and social burden. In 2010, latest estimates suggest 219 million cases with an uncertainty range of (154 million, 289 million) cases globally. There were 660 000 deaths due to malaria; with 90% of deaths occurring in Africa and most cases and deaths occurring in sub-Saharan Africa [1]. Malaria has been recognized as a disease of poverty with vulnerable groups facing several barriers to access for antimalarial interventions [2], [3]. South Africa, having experienced a sharp decline in malaria cases since the last epidemic in 2000, already meets the pre-elimination phase criteria set out by the World Health Organisation (WHO) (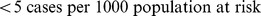) and has been ear-marked to achieve elimination by 2018 [4]. Mpumalanga is one of three provinces in South Africa where malaria transmission still occurs. Malaria in Mpumalanga is seasonal, starting with the first rains in October, peaking in January and remaining high till May; yet transmission is still unstable and prone to sporadic outbreaks. As government begins to intensify efforts and commit scarce resources towards malaria elimination, there is a need to understand patterns in transmission so that efforts may be targeted appropriately. Mathematical modeling is increasingly being used to test policy interventions so as to determine their impact on simulated transmission before implementing the intervention in the field. Understanding the nature of the seasonality of transmission will enable better mathematical modeling and this may lead to better allocation of scarce resources and ultimately a greater impact on malaria. This paper aims to explore the seasonality of malaria cases in the Mpumalanga province as part of a larger project in mathematical modeling of malaria transmission and the impact of antimalarial interventions. We analyse data on reported treated malaria cases from 2002 to 2012. In particular, this paper explores the temporal and geographic behavior patterns as well as potential drivers behind these patterns.

**Figure 1:**
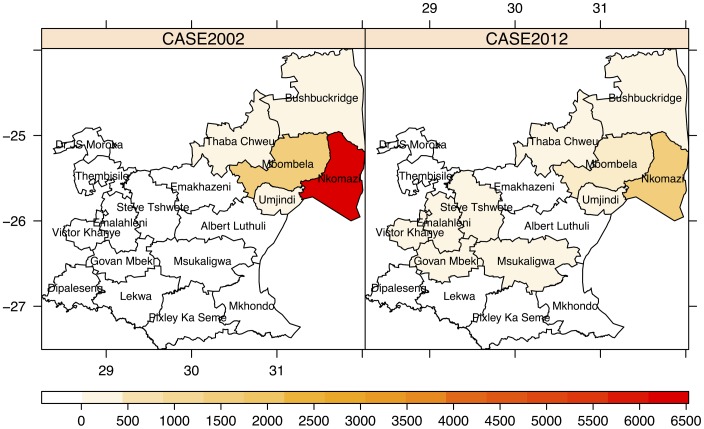
The number of reported treated cases are depicted for all the municipalities in Mpumalanga Province for 2002 and 2012.

Malaria control in South Africa and Mpumalanga is well documented [5]–[11]. Malaria is distributed mainly in the low-lying areas bordering Swaziland and Mozambique, with a favourable climate for malaria transmission. Nkomazi, Bushbuckridge, Mbombela, Umjindi and Thaba Chewu local municipalities (part of the Ehlanzeni district) are the districts mostly affected by malaria (Figure 1). Kruger National Park and surrounding lodges have been incorporated into the Bushbuckridge and Mbombela municipalities and comprise 1.7% of all cases in the study period. Transmission is most intense in the municipalities bordering Mozambique. Table 1 shows the incidence rates for all cases treated in the province by local municipality using population estimates from Statistics South Africa [12]. While there is a sharp decrease in incidence in the province between 2002 and 2012, this decrease is not consistent over the local municipalities. Plasmodium falciparum is the predominant parasite that is transmitted primarily by the Anopheles arabiensis vector [13]. As malaria is a notifiable disease in South Africa, malaria information systems have been developed to record all malaria cases that are Plasmodium positive through a rapid diagnostic test or by slide microscopy [14]. Active case detection, a strategy of following up notified cases to verify the source of infection thereby allowing further screening and treating of symptomatic people, is also employed in the province. Owing to an increase in gametocyte carriage following Sulphadoxine-Pyremethamine (SP) treatement in 2002, the province made a switch to SP-artesunate in 2003, followed by Artemether/Lumefantrine (AL) in 2006 [14]–[16]. Vector control in the province includes indoor residual spraying (IRS) using primarily dichorodiphenyltrichloroethane (DDT) and larviciding at identified breeding sites [6]. There are also two collaborative cross-border initiatives aimed at reducing incidence in participating countries. The Trans-Limpopo Malaria Initiative targets the Matabeleland South province of Zimbabwe and Limpopo province in South Africa, and the Lubombo Spatial Development Initiative targets eastern Swaziland, Maputo and Gaza provinces in Mozambique and Kwazulu-Natal province in South Africa [11]–[17].

**Table 1:** Malaria Incidence Rates.

Area	2002 Cases	2002 Incidence per 1000 people	2012 Cases	2012 Incidence per 1000 people	% Change in Cases
Mpumalanga	7933	238	2745	68	−65%
Nkomazi Municipality	6100	1793	1540	394	−75%
Mbombela Municipality	1316	270	637	108	−52%
Bushbuckridge Municipality	282	56	312	58	11%
Umjindi Municipality	177	320	120	172	−32%
Thaba Chewu Municipality	34	41	23	23	−32%

Table showing malaria incidence and the change in cases in Mpumalanga Province and the municipalities in Ehlanzeni District.

This paper will examine the seasonal pattern of malaria cases using time series methods and explore the relationship between cases and three potential drivers: rainfall, geography and source of infection. The relationship between malaria transmission and climate has been examined extensively [18]–[22] and Ngomane and de Jager [6] showed that rainfall was the only significant climatic factor in this Mpumalanga dataset examined between 2001 and 2009. Migration and its impact on malaria transmission are affected by both the primary physical location where the infection resides as well as the source of the infection. Migrant human populations may affect transmission in two key ways. Firstly, people from areas of low malaria transmission move to areas of high transmission and having little or no immunity become infected. In the second case, people from areas of high transmission may harbour parasites and transmit these when they move to areas of low transmission. Having partial or full immunity, they may not exhibit clinical symptoms and hence become hidden reservoirs of infection [23]. As Mpumalanga shares a border with Mozambique (which has a higher malaria prevalence) and Swaziland, it is of interest to assess both the geography and source of infection as potential drivers of the seasonality of cases in Mpumalanga.

## Methods

### Ethics statement

This study is an analysis of secondary data. Ethical approval for use of the notification data was obtained from the University of Cape Town Human Research Ethics Committee and the Mpumalanga Department of Health. Written consent was given by the patients for their information to be stored in the hospital database and used for research.

### Data

To explore malaria cases, one would require data on both the treated and untreated cases to capture the populations that are unable to access treatment, as well as the asymptomatic population. As asymptomatic people would not usually present for treatment at a facility, it is rarely the case that such data is available. The only data available in Mpumalanga are those cases that presented for treatment at a public health facility and actively detected cases where index cases are followed up in home and malaria diagnostic tests are performed on nearby households. This data is sourced from provincial Integrated Malaria Information System (IMIS) under the management of the Malaria Elimination Programme of the Department of Health. The data also included the following information: date of diagnosis, age, gender, mortality indicator, drug, facility name, administrative municipality, source of infection (country, province, locality) and place of residence (country, province, locality). Source of infection has been determined for all cases in the province, whereby a case is classified as imported if the patient travelled to a malaria-endemic area in the past month or if there is no evidence of local transmission(vectors or cases within 500 m radius of the place of residence) [17]. Provincial border changes in the study period required the addition of malaria cases from Bushbuckridge pre-2006 (then part of Limpopo province) and the exclusion of cases from Limpopo post-2006. Source of infection data was not always available for this additional data, which comprises only 3.4% of all cases. Monthly case data from the province of Maputo in Mozambique was obtained for the study period from the Mozambique National Ministry of Health. The analysis of the relationship between rainfall and cases required obtaining monthly rain data for the Ehlanzeni district from the South African Weather Services for the study period.

### Data Analysis

Case data was compiled in a weekly format in Stata 11 and analysed using time series methods [24]. As case data usually exhibits noise, it is often difficult to draw conclusions on seasonal patterns based on the case data itself and hence there is a need to extract the seasonal pattern from the data. In particular, Seasonal decomposition of Time series by LOESS (STL) method of extracting components was used to assess the seasonal pattern of the data. Decomposition methods generally separate a time series into a trend (moving average) component, a seasonal (systematic temporal variation) component, a cyclical (repeated non-periodic fluctuations) component and a residual (remainder; irregular random) component [25]. STL is one such decomposition method where LOESS (LOcally Estimated Scatterplot Smoothing) is applied iteratively to the observations in moving time windows to filter the time series in a way that results in estimates of trend and seasonality that are robust to aberrant behaviour in the time series (Cleveland, 1990).

The relationship between weekly rainfall and weekly reported treated cases is assessed through a cross-correlation analysis of the pre-whitened series (removing all autoregressive, moving average, integrated components and seasonal dynamics to achieve a series that has a zero mean, constant variance and no correlation between observations at different points in time) to determine if any lagged values of rainfall are significantly correlated with reported treated cases. The series are pre-whitened using Seasonal AutoRegressive Iterated Moving Average (SARIMA) methods. Data analysis is performed in R v3.0.1 [26].

## Results

The analysis for the case data is presented first followed by an assessment of the relationship between cases and rainfall, cases and physical geography and thirdly, cases and source of infection.

### Case Data

**Figure 2:**
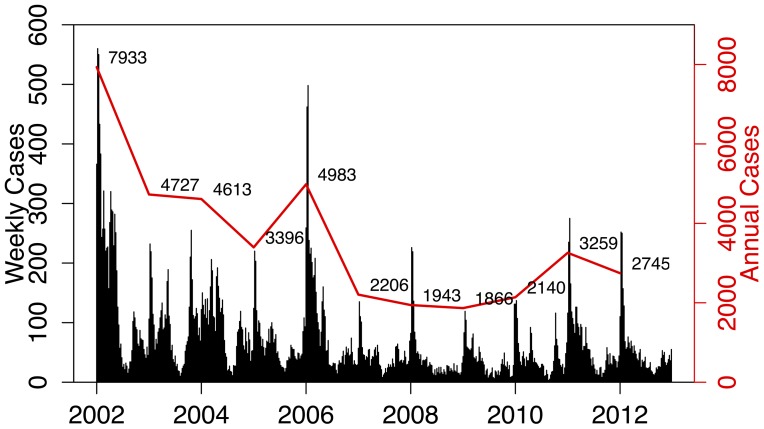
The weekly and annual reported treated malaria cases in Mpumalanga Province between 2002 and 2012.

**Figure 3:**
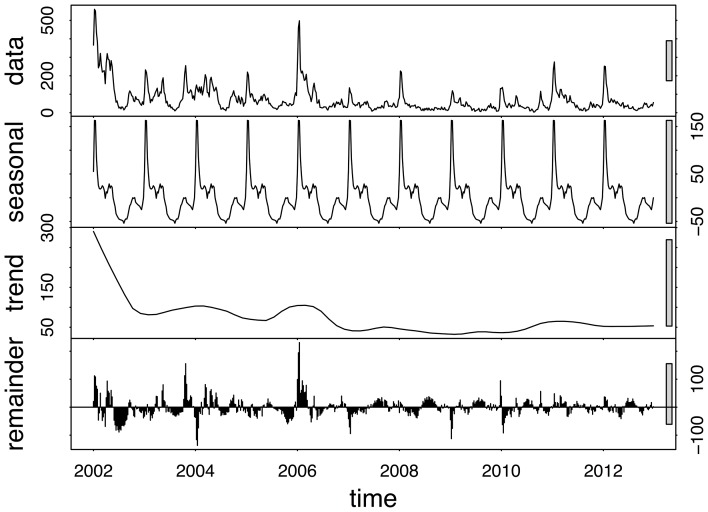
STL decomposition on reported treated malaria cases in Mpumalanga Province between 2002 and 2012.

**Figure 4:**
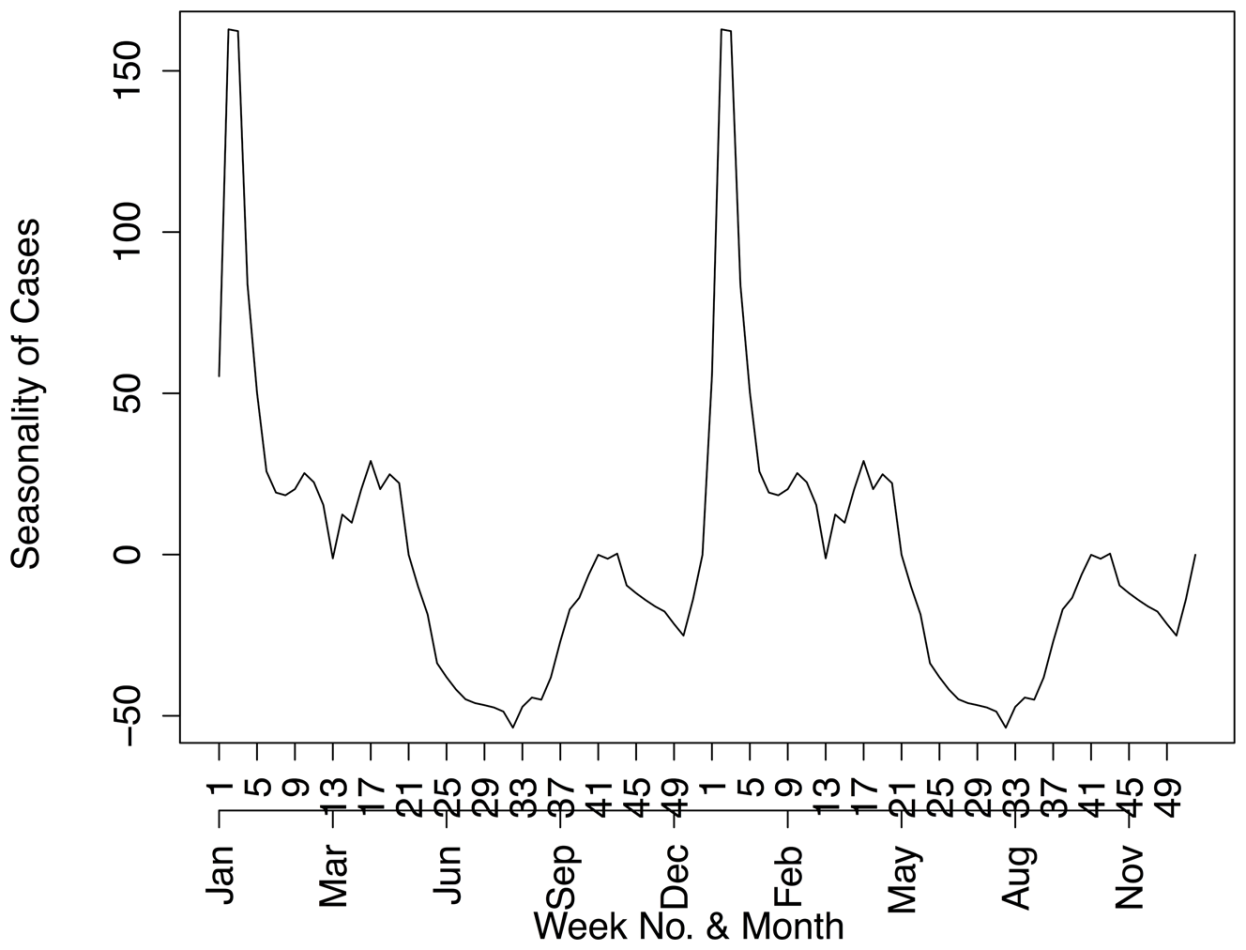
Two year seasonal trend of reported treated malaria cases in Mpumalanga Province between 2002 and 2012.

Figure 2 shows the annual and weekly counts of reported treated cases over the period 2002 to 2012. In this period, there were 40650 reported malaria cases (imported and local) and 256 deaths due to malaria in the province. Efforts to scale-up IRS in the province and the switch to artemisinin-based combination therapy have contributed to the 65% decrease in reported cases since 2002. Annual reported figures for malaria remained consistently low after the surge of cases in 2006, but the 2011 and 2012 counts are considerably higher than preceding years. The total time series of reported treated cases is decomposed into trend, seasonal and random components using the STL decomposition. The crude data series in Figure 3 (top panel) appears at face value to have pronounced seasonal pattern from 2002 to 2006, but this becomes less distinct post-2006. The STL decomposition however extracts the general seasonal pattern that is unlike standard single-peaked sinusoidal annual seasonal patterns. The seasons are characterized by very short periods of no/low level of reported cases and a triple peak in reported cases between September and May. The trend component shows the steady decrease in reported cases since 2002, with a sharp increase in 2006 and a more moderate increase recently in 2011. The remainder component is the component of the time series that remains after the trend and seasonal components have been removed from the series. It can be used to detect anomalies in the data; points in the data set where the reported cases series has deviated from the trend and seasonal patterns. For example, the seasonal component suggests that the second peak is usually higher than the first peak; yet in 2004 the upward spike in the remainder component highlights that the first peak of the season was higher than the second peak. The remainder component also depicts the sharp increase in the series in 2006. Figure 4 focuses on 2 years so as to highlight the timing of the season. It can be seen that the first peak in reported cases occurs approximately between weeks 38 and 44 (mid-September to end-October), the second peak occurs between weeks 52 and 4 (end-December to end-January) and the third peak occurs between weeks 14 and 22 (start-April to end-May).

### Cases and Rainfall

**Figure 5:**
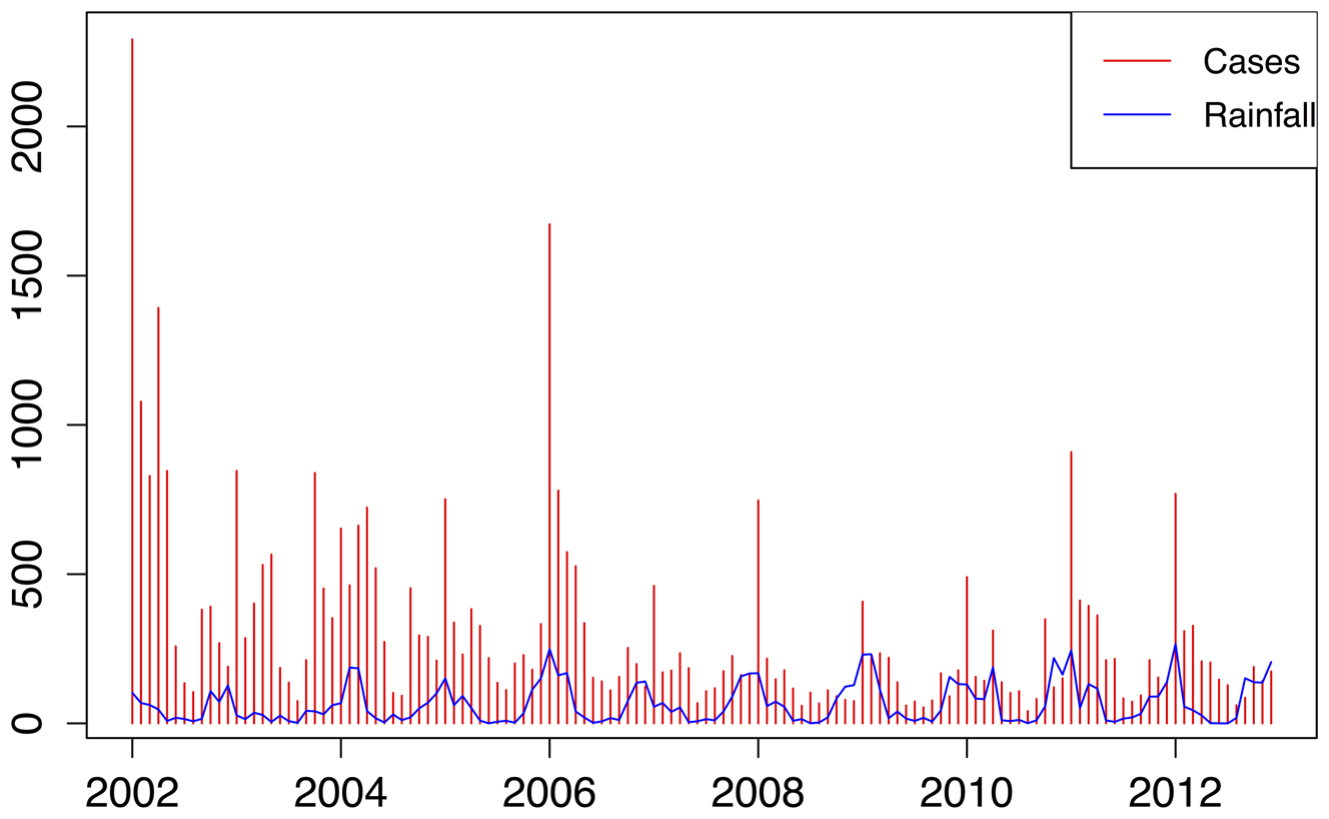
Weekly malaria cases and monthly average rainfall in Mpumalanga Province between 2002 and 2012.

**Figure 6:**
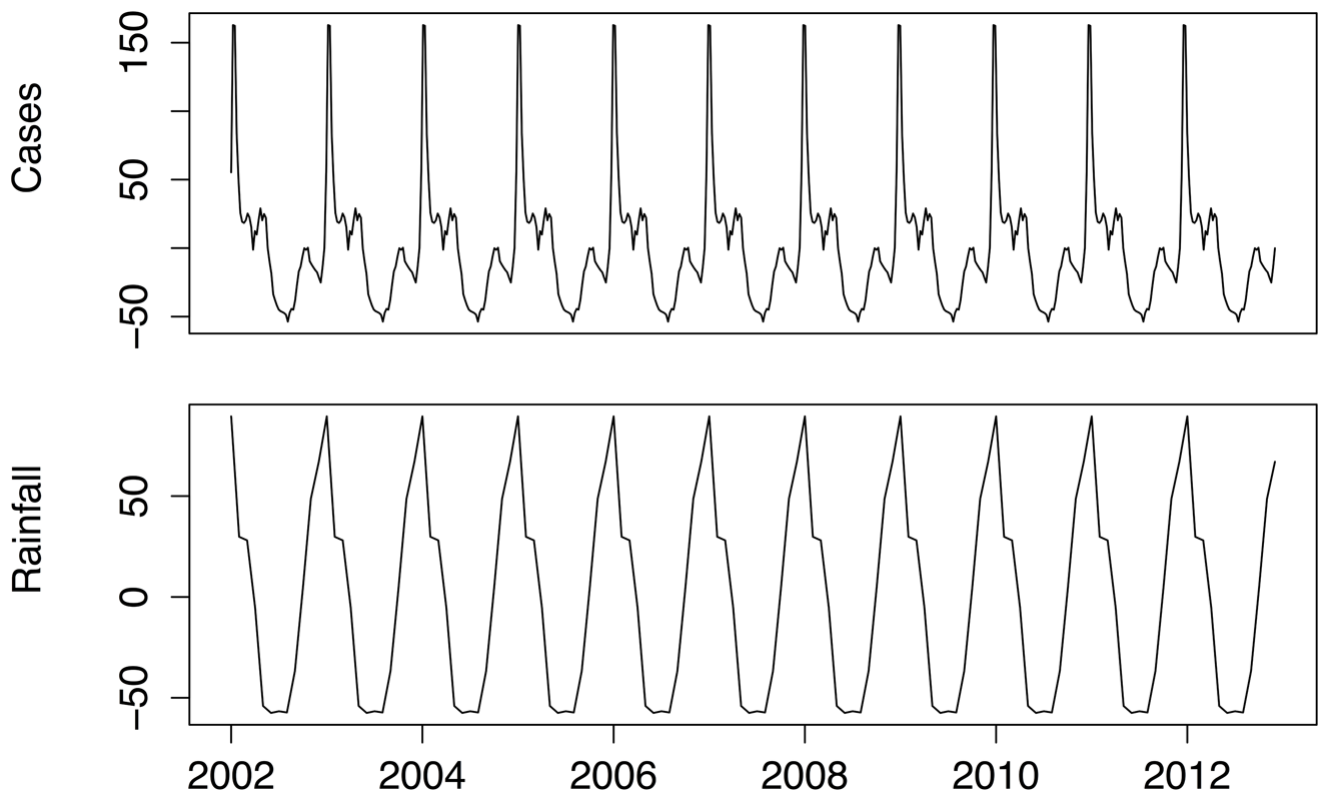
Seasonal trend of weekly malaria cases and monthly average rainfall in Mpumalanga Province between 2002 and 2012.

Ngomane and de Jager [6] assessed the relationship between malaria incidence and rainfall, relative humidity and minimum and maximum temperature in an ARIMA framework and found rainfall to be the only significant climatic factor. The seasonal pattern of monthly rainfall coincides with the height of the malaria season early on in the time series but appears to lag the season post 2006 (Figure 5). Further the period of higher rainfall pre-2006 appears to correspond with the higher number of cases reported (and treated) in 2006, but this does not appear to be the case for the higher number of cases in 2011 and 2012. The STL seasonal decomposition of rainfall reveals a single peak that coincides generally with the main peak of the case data (Figure 6). This analysis is pursued as epidemiologically, rainfall has a delayed impact on malaria incidence due to the incubation and latent phases of the parasite in the vector and the host. Of interest is thus to analyse the correlation between lagged rainfall values and malaria incidence. A cross correlation analysis is performed between lagged rainfall and the case data. The case and rain series are pre-whitened to avoid spurious correlations. The series are square-rooted first to stabilise the variance and then the Box-Jenkins modeling algorithm is used to select the resultant SARIMA(1,1,1)(0,1,1)12 model [27]. This model was selected as it had the lowest Akaike Information Criterion value among all models and made epidemiological sense. There is no autocorrelation or partial autocorrelation present in the residual series. It was found that there is a significant positive correlation of 0.21 between values of monthly rain at lag 1 (4 weeks) and monthly cases (assessing negative lags, as lagged cases cannot be used to predict rainfall).

### Cases and Source of Infection

**Figure 7:**
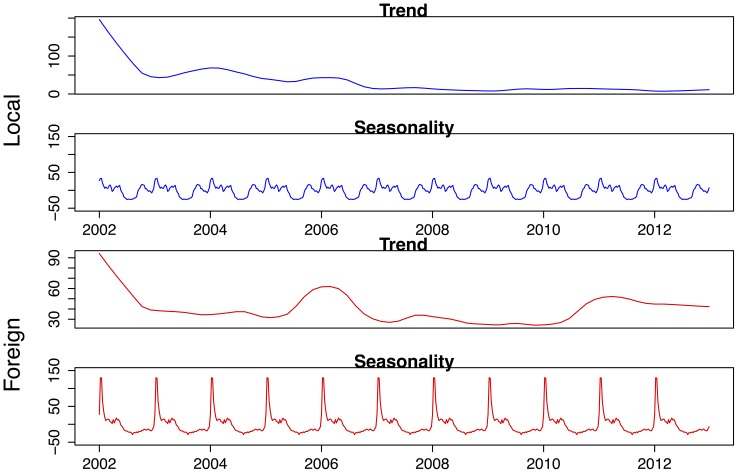
Seasonality and trend components of an STL decomposition of locally and foreign-sourced malaria cases in Mpumalanga Province between 2002 and 2012.

**Figure 8:**
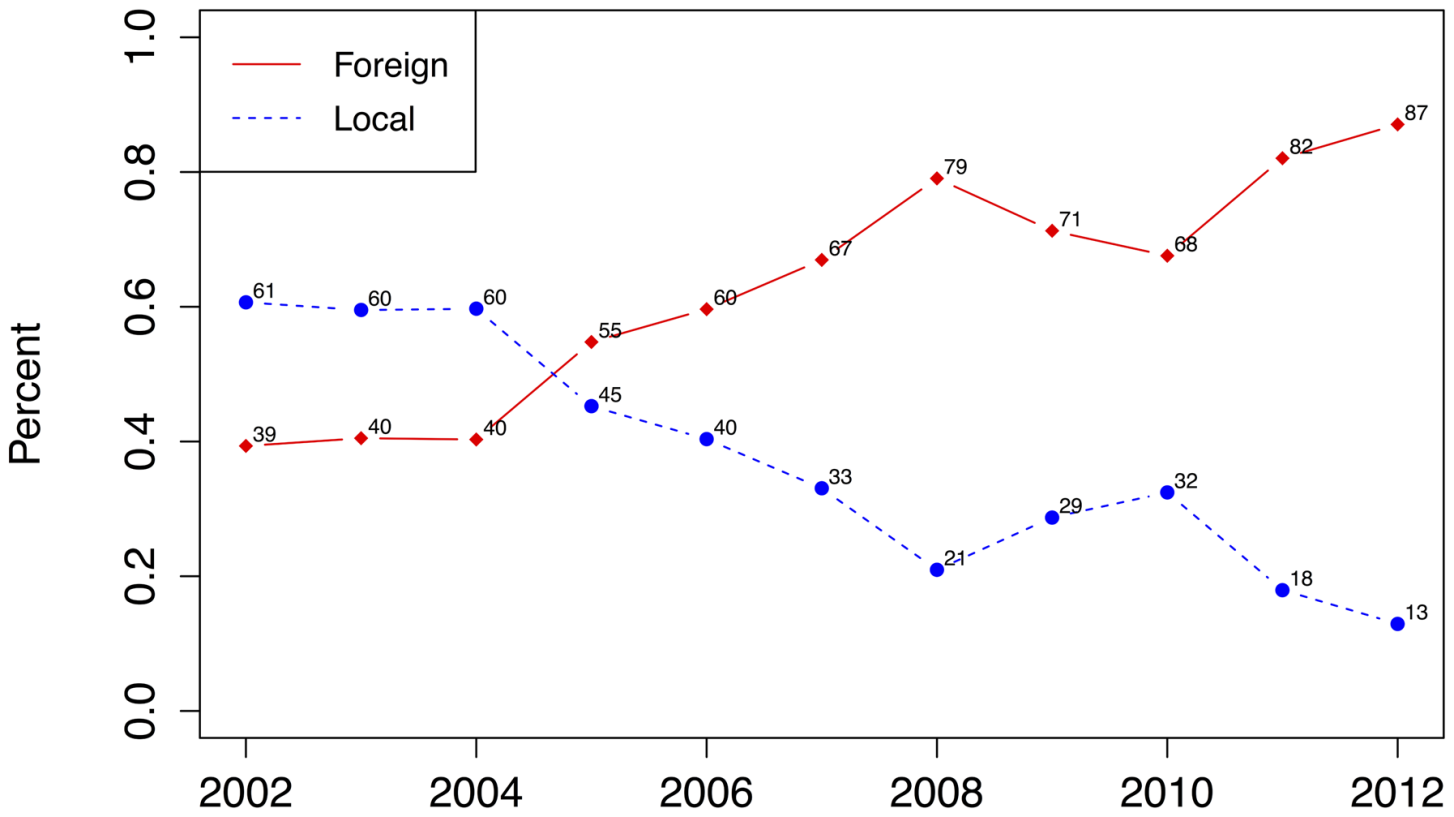
Percentage plot of locally and foreign-sourced malaria cases in Mpumalanga Province between 2002 and 2012.

To further explore the drivers behind the three-peaked seasonal pattern of the case data, foreign and locally sourced case counts are assessed. Each reported case is assessed to determine if the infection is obtained from a local source (within South Africa) or from a foreign source (outside South Africa). Figure 7 compares the trend and seasonal components for locally and foreign-sourced infections for all reported treated cases. Locally sourced infections have been on a steady decrease since 2002, remaining at very low levels while foreign-sourced infections show an increase in 2006, with a further increase in 2010 and has remained at this level since. The seasonal pattern of the locally-sourced infection mimics the triple-peak characteristic of the total series while foreign-sourced infections appear to contribute mostly to the second and third peak of the malaria season, and not the first peak of the total series (Figure 3: 2nd panel). The annual distribution of foreign and locally sourced infections has changed considerably during the study period (Figure 8). Foreign-sourced infections have dominated locally sourced infections since 2005, and this proportion has been increasing since. At the end of 2012, 87% of all reported treated cases had a foreign-source.

### Cases and Physical Geography

**Figure 9:**
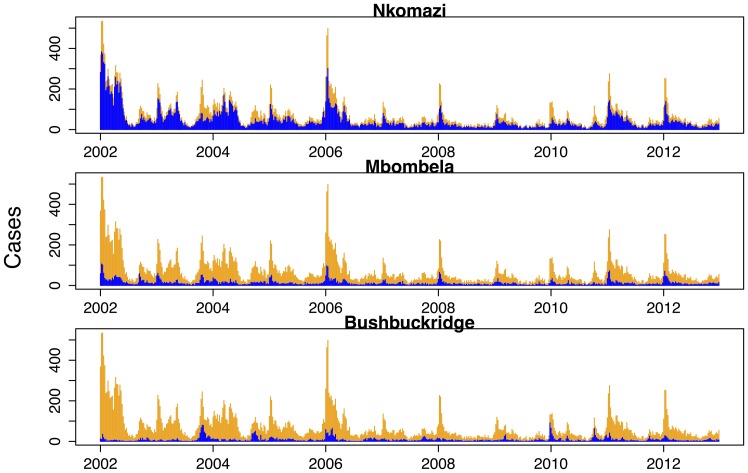
Weekly malaria cases in Mpumalanga Province (orange) and Nkomazi, Mbombela and Bushbuckridge Municipalities (blue) between 2002 and 2012.

**Figure 10:**
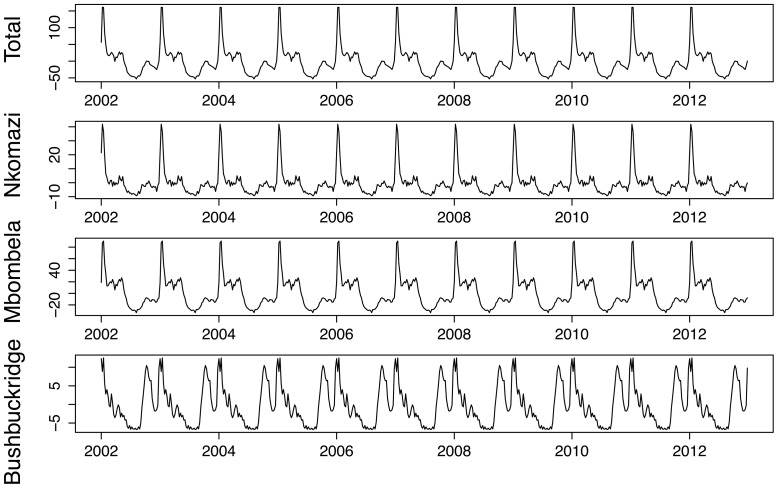
Seasonal trend of weekly malaria cases in Nkomazi, Mbombela and Bushbuckridge Municipalities between 2002 and 2012.

**Figure 11:**
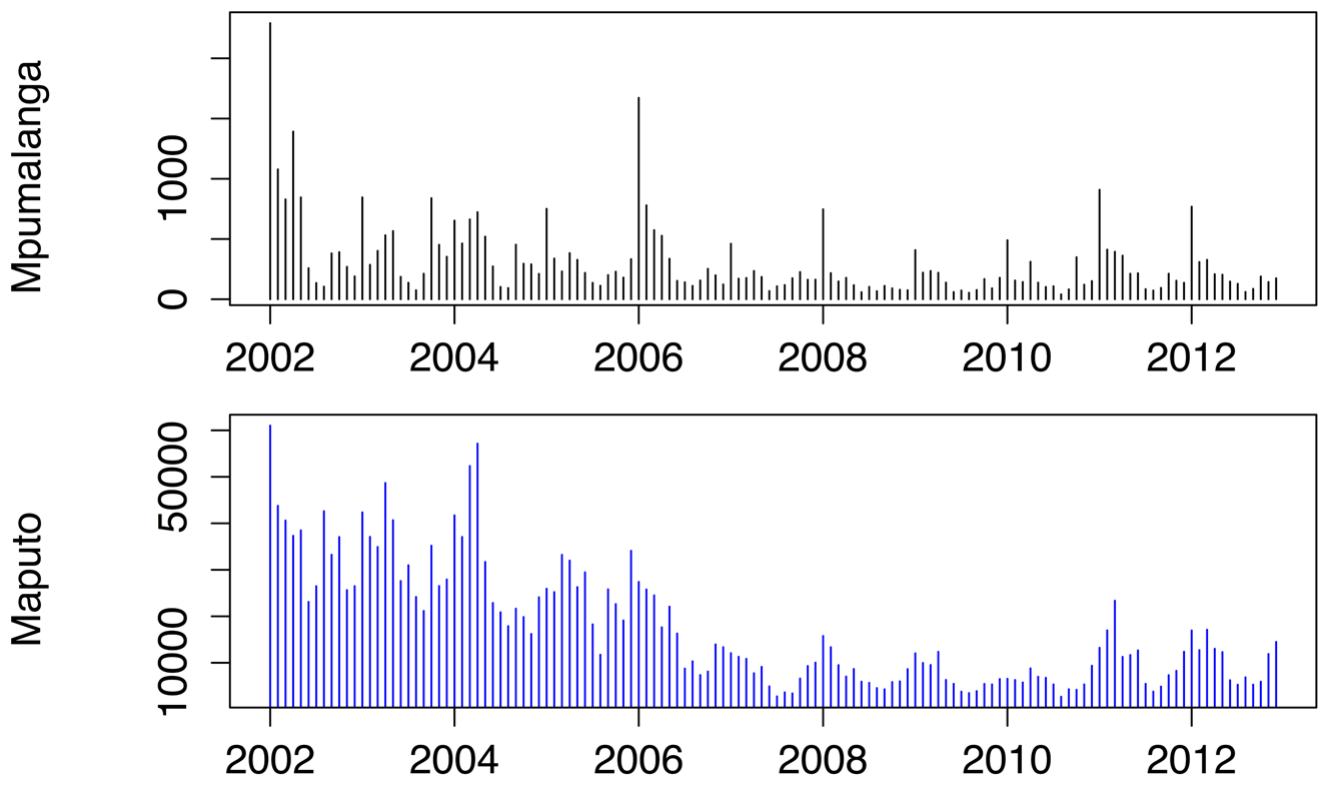
Monthly malaria cases in Mpumalanga and Maputo, Mozambique between 2002 and 2012.

**Figure 12:**
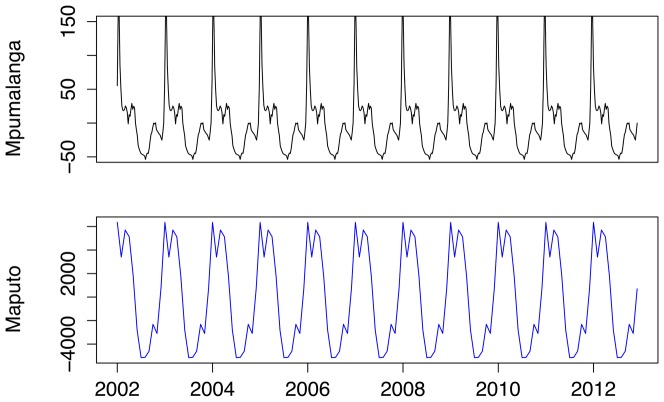
Seasonal trend of monthly malaria cases in Mpumalanga and Maputo, Mozambique between 2002 and 2012.

Physical geography is assessed with regards to location within Mpumalanga (local municipalities) and the pattern of cases in the contiguous Maputo province in Mozambique. Nkomazi, Mbombela and Bushbuckridge have consistently been the municipalities with the highest number of reported treated cases. Figure 9 shows the weekly counts of reported treated cases for each of the municipalities against the backdrop of the time series for all municipalities. Nkomazi is clearly home to most of the reported malaria compared to Mbombela, and while Bushbuckridge on its own, does not usually contribute greatly to total malaria, its contribution is sporadic with unusually large number of cases in 2004, 2006 and 2010–2012. Looking at the seasonal components extracted from these municipalities, one can see that both Nkomazi and Mbombela municipalities exhibit the triple-peak pattern as seen for the total series. Reported treated cases in Bushbuckridge municipality also exhibit a triple-peak but the first and second peaks are approximately the same size unlike in the other municipalities (Figure 10). Of the 40650 cases analysed, 41% of cases were sourced in South Africa and 54% sourced from Mozambique (the remaining 5% being sourced from other African and Asian countries). Figure 11 depicts the monthly case profile for Maputo province and Figure 12 shows the seasonal profile for the two provinces where the peak of the malaria season in Maputo corresponds to the second and third peaks of the Mpumalanga season. This is consistent with the seasonal pattern of foreign-sourced infections presented earlier.

## Discussion

Malaria transmission in Mpumalanga is characterized by a triple-peak in the season where the first peak occurs in September/October, the second (and also main) peak occurs in January and the third peak occurs in April/May. Assessing the STL seasonal components of the source and geographical location of infection shows that the first peak is driven by mainly locally-sourced infections and the second and third peaks are driven mainly by foreign-sourced infections. This is supported by the Maputo data where the peak of the season in Maputo corresponds to the second and third peak of the Mpumalanga season. Geographically, Bushbuckridge, the northern most municipality, contributes specifically to the first and second peaks, while the other two municipalities studied contribute equally to all three peaks. Monthly rainfall lagged at 1 month was found to be significantly associated with monthly cases, yet the seasonal pattern of rainfall does not appear to characterize the triple peak pattern of the case data. The decline of local cases in Mpumalanga may be a reason for this, where cases from a foreign source are more likely to be affected by rainfall in the source area (e.g. Mozambique) than local rainfall. If the increase in the proportion of foreign-sourced cases persists, rainfall may become even less of a driver for malaria. Having reduced its malaria burden significantly since 2002, South Africa has embraced a malaria elimination target of 2018 [4]. To achieve this, suites of malaria control interventions will be deployed in Mpumalanga and other provinces affected by malaria. The likely impact of these interventions may be measured through mathematical modeling. The MalERA Consultative Group on Modeling has recognized the contribution mathematical modeling can make to the elimination of malaria globally and has developed a framework of priority areas for modeling to assess and inform: optimal resource allocation, strategies to minimize the evolution of drug and pesticide resistance, new tools to interrupt malaria transmission, combinations of tools, coverage targets and expected timelines to achieve goals as well as to assess operational feasibility with respect to costs and human resource capacities [28]. Using mathematical modeling to adequately represent malaria transmission requires knowledge on the drivers of the geographic and temporal trends in malaria transmission and this in turn may lead to finding malaria control strategies that target these drivers directly rather than strategies that may generally control transmission but not interrupt it. The primary interest of mathematical modeling in this setting is to provide practical guidance to malaria programme managers on how to conduct more efficient and effective control and elimination activities. This paper assesses the seasonal trend of cases for different sub-groups of the population, based on the source of infection and geography. If an elimination activity such as the scale-up of larviciding is under consideration, knowing the seasonal pattern of locally sourced cases (larviciding impacts locally sourced cases directly) allows managers to optimally time the larviciding activities with the breeding patterns of the mosquitoes. While malaria elimination requires the reduction to zero of locally sourced cases only, interventions aimed at reducing foreign sourced cases can reduce onward transmission. Knowing the seasonal pattern of foreign sourced cases allows programme managers to optimally time interventions such as mass screen and treat campaigns at border posts with travel/migration patterns. Understanding the seasonal differences between managerial districts like municipalities can also assist with optimal allocation of drugs and staff. These are some of the many practical uses of mathematical modeling in health management. The rising percentage of imported or foreign-sourced cases suggests the need for intensive monitoring. The second and third peaks of the season (which comprise mainly foreign-sourced cases) correspond to holiday seasons (Christmas and Easter). As the majority of cases are sourced from Mozambique, there is a need to intensify cross-border collaborations now and even more so after elimination has been achieved in South Africa. This increase in foreign-sourced cases also highlights the importance of including migration in mathematical models of malaria transmission to obtain realistic estimates of the impact of malaria control and malaria-elimination focused strategies.

There are two main limitations of this study. Firstly, the case data available is for those who were treated at public health facilities and some private practices and no malaria case information is known for others treated in the private sector and the untreated population. The untreated population may represent vulnerable groups such as the very poor who are often unable to access treatment and illegal immigrants who may avoid the health system. If the latter group has developed immunity to malaria and is therefore asymptomatic, they could be responsible for unknowingly transmitting the disease. Secondly, routine surveillance data are also subject to errors in reporting that may bias the analysis. The collection of notification data is still recommended by WHO with active/passive case detection assisting in the identification of additional asymptomatic cases. A provincial census or a mass screen and treat campaign could provide cross-sectional data on the untreated population, but this is both costly and non-informative temporally.

## Conclusion

Mpumalanga province in South Africa has experienced a 65% decrease in reported treated cases since 2002. In this time, the percentage imported cases of this total has increased from 39% to 87%. Using weekly reported treated cases, the geographic and temporal trends in malaria transmission was explored to reveal an atypical triple-peak pattern in cases with a short period of few cases. This seasonality was explored in relation to rainfall, source of infection and geographic location. A cross correlation analysis revealed that one month lagged rainfall was significantly associated with reported treated cases, but the seasonality of rain did not appear to explain the unusual pattern of malaria cases. Analysis of the source of infection revealed that local cases contribute to all three peaks but foreign cases contribute primarily to the Christmas and Easter peaks. Geographically, Bushbuckridge municipality (northern most municipality) had a greater relative contribution of cases in the first (September/October) peak. Malaria transmission in Mpumalanga may be low, but it is also unstable and a change in climate and the source of infection may lead to a spike in infection and generally a modification of the incidence pattern. These findings may be useful as they enhance the understanding of the current incidence pattern and can be incorporated in mathematical models that enable one to predict the impact changes in these drivers will have on malaria transmission. Further, as mathematical modeling is being used to assess timeframes for malaria elimination and the potential impact of elimination-focused interventions, understanding the seasonal trends of malaria is key to designing a targeted temporal and spatial approach that can be applied in resource-scarce settings.
